# Failed Thumb Basal Joint Arthroplasty: An Analysis of Common Etiologies Leading to Reoperation

**DOI:** 10.1016/j.jhsg.2024.12.004

**Published:** 2025-01-31

**Authors:** Adina Harri, Emily J. Harman, Jennifer Chickering, Lance G. Warhold, Vincent D. Pellegrini

**Affiliations:** ∗ Geisel School of Medicine at Dartmouth, Hanover, NH; † Dartmouth Hitchcock Medical Center, One Medical Center Drive, Lebanon, NH

**Keywords:** Basal joint, CMC osteoarthritis, Revision surgery, Trapeziometacarpal instability, Trapeziometacarpal joint

## Abstract

**Purpose:**

Hand osteoarthritis contributes considerably to functional disability, and the basal joint is the most common site treated surgically. Procedure choice is largely based on surgeon preference, and causes of primary basal joint arthroplasty failure and prevalence of revision are poorly defined. We seek to understand the etiologies of revision basal joint surgery.

**Methods:**

Retrospective medical record review identified 26 reoperations in 24 thumbs in 23 patients by two senior hand surgeons following primary basal joint arthroplasty performed for osteoarthritis from 2014 to 2022. Chart review yielded demographic and historical information, surgical technique of index and revision procedures, presenting symptoms, time to reoperation, and intraoperative revision findings. Radiographic measures included prerevision thumb metacarpal subsidence, radial subluxation, scaphoid impingement, and untreated scaphotrapezoid arthritis.

**Results:**

All patients complained primarily of pain on presentation, followed by impaired function (n = 11) and dorsal thumb dysesthesias (n = 2). Physical findings included metacarpophalangeal joint hyperextension (n = 12), axial thumb instability (n = 10), and inability to flatten the palm (n = 5); the latter occurred in four of seven thumbs having index TightRope or swivel-lock procedures and one thumb having ligament reconstruction and tendon interposition with a swivel-lock device. Six patients (25.0%) had “other” pathologies including carpal tunnel syndrome, palpable foreign body, capitate subluxation, and absent EPL function. Radiographs revealed untreated scaphotrapezoid arthritis (18), radial metacarpal base subluxation (18), proximal thumb metacarpal migration (17), and trapezial space height < 5 mm (16). Intraoperative revision findings included proximal thumb metacarpal migration (12), untreated scaphotrapezoid arthritis (9), foreign body granuloma (6), metacarpophalangeal joint hyperextension (5), impinging osteophytes (4), scaphoid-metacarpal impingement (3), and suture anchor pull-out (2).

**Conclusions:**

The most common pathologies encountered during revision basal joint arthroplasty include trapeziometacarpal instability, unrecognized scaphotrapezoid arthritis, and untreated metacarpophalangeal joint hyperextension. Dysfunction resulted from iatrogenic inability to flatten the palm associated with use of swivel-lock and TightRope anchor devices to stabilize the thumb metacarpal.

**Type of study/level of evidence:**

Differential Diagnosis/Symptom Prevalence Study IV.

The opposable human thumb requires functional palmar abduction as well as strength and is susceptible to osteoarthritis (OA) that compromises the ability to grasp large objects, turn a key, and drink from a glass with one hand. Hand OA is the second most prevalent anatomic site, is a notable contributor to years lived with disability, and most commonly involves the thumb “basal” joint, making it the most common site of surgery for OA in the upper limb.^1^, ^2^, ^3^, ^4^, ^5^, ^6^ Although the “basal joint” includes four trapezial articulations, the trapeziometacarpal joint is most commonly the primary site of pathology. Notwithstanding its prevalence, there is no consensus about which operation is most effective.^7^, ^8^, ^9^, ^10^, ^11^, ^12^, ^13^, ^14^, ^15^, ^16^, ^17^, ^18^ Surgical technique is, therefore, discretionary and often based on surgeon preference.

Ligament reconstruction and tendon interposition (LRTI) arthroplasty is the most commonly performed procedure, some newer techniques claim faster recovery, and most basal joint procedures are successful.^19^, ^20^, ^21^, ^22^, ^23^ Nevertheless, some patients inevitably experience complications and undergo revision surgery. Although the prevalence of revision basal joint arthroplasty (BJA) is largely unknown, its frequency is estimated at 2.5% to 3%.^23^, ^24^, ^25^, ^26^ Systematic analysis of failed primary BJA and guidance for revision surgery is limited.^27^, ^28^, ^29^

Accordingly, we studied patients who had undergone revision arthroplasty and analyzed presenting symptoms and signs, objective radiographic parameters, and intraoperative findings noted at revision surgery. We hypothesized that thumb metacarpal instability, untreated scaphotrapezoid arthritis, and metacarpophalangeal (MCP) joint hyperextension would contribute to persistent symptoms and inform strategies for revision surgery. We neither studied nor commented on the outcomes of revision BJA; rather, we analyzed patients who had undergone revision BJA to determine common etiologies of failure.

## Materials and Methods

We performed a retrospective review of consecutive patients undergoing operation for failed primary BJA by two senior hand surgeons at our institution from 2014 through 2022. Institutional review board review determined that the work warranted exempt status. A medical record search using Current Procedural Terminology and International Classification of Diseases-Ninth and Tenth Revision codes yielded 23 patients with 26 reoperations performed in 24 thumbs. One patient underwent a single revision on each thumb, and two patients underwent a second revision on one thumb. Four index procedures were performed by one of us, whereas all others were performed elsewhere and referred specifically for consideration of revision surgery.

Chart review yielded demographic information including medical history, surgical technique of index and revision procedures, revision indications based on preoperative clinical notes, time to reoperation, and intraoperative findings at revision surgery. Radiographic assessment of prerevision proximal thumb metacarpal migration (subsidence), radial subluxation, scaphoid impingement, and untreated scaphotrapezoid arthritis was performed specifically for this review.

### Radiographic assessment

The most recent posteroanterior hand radiograph prior to revision was used for all analyses. Prerevision radiographs were not available for one thumb. Standardized measurements were performed independently by two reviewers; when necessary, a third blinded reviewer served as tiebreaker.

Proximal thumb metacarpal migration was indicated by discontinuity of the line connecting the subchondral base of the index and thumb metacarpals, that is, Shenton’s line of the thumb (Fig. A, C).

**Figure:**
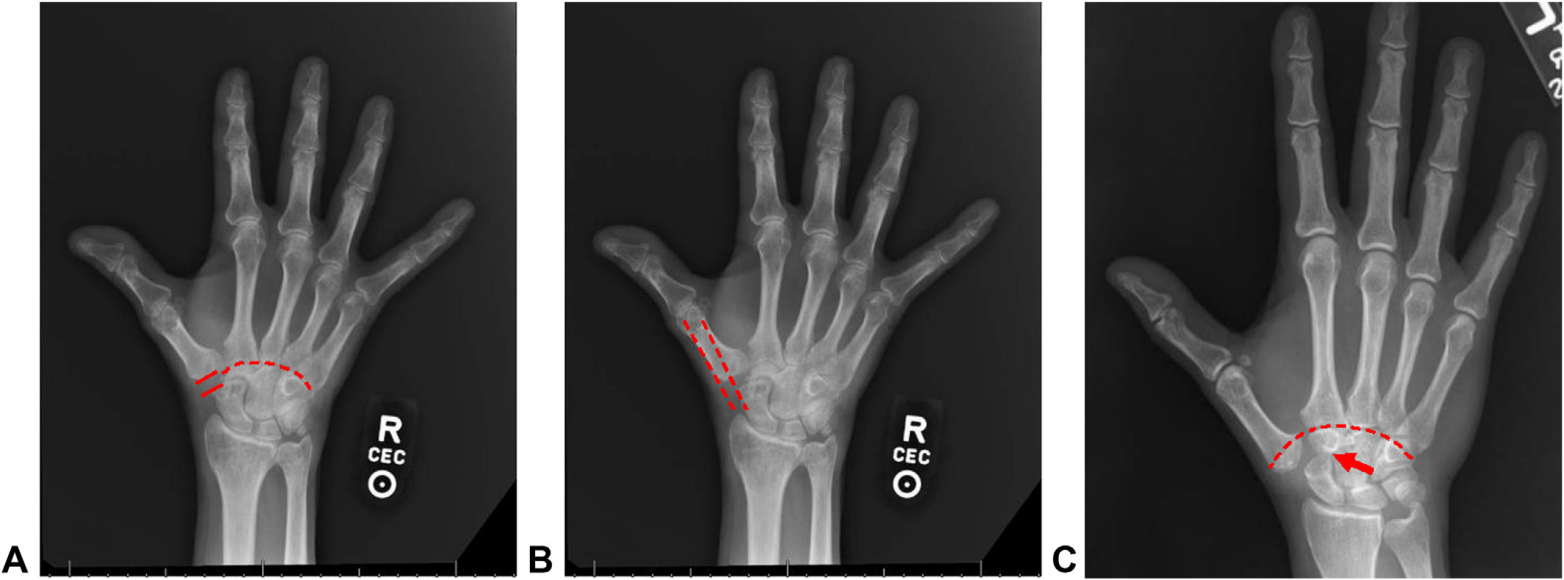
Measurement of radiographic features in thumbs undergoing revision arthroplasty. Metacarpal instability is reflected in trapezial space height (A), percent radial subluxation of the first metacarpal base (B), and migration of the thumb metacarpal base (dashed lines in A, C). Trapezial space height is measured from the base of the thumb metacarpal to the distal tip of the scaphoid. Radial subluxation of the thumb metacarpal is measured by extending a line along the radial cortex of the thumb metacarpal shaft, translating it ulnarly until meeting the border of the scaphoid, and dividing this distance by the width of the thumb metacarpal base. Migration of the thumb metacarpal base is measured by Shenton’s line, which is depicted as a continuous (dashed line, A) or discontinuous (dashed line, C) line of curvature connecting the proximal limit of the index and thumb metacarpals. Scaphotrapezoid arthritis (C, arrow) is evidenced by subchondral sclerosis and osteophytes. In the two leftmost radiographs, R refers to right-sided and CEC refers to using radiographic quality criteria developed by the Commission of European Communities (CEC).

The degree of radial subluxation of the thumb base relative to the scaphoid was determined by extending a line parallel with the radial cortex of the thumb metacarpal shaft and translating that line ulnarly to meet the radial border of the scaphoid (Fig. B). The ratio of the translated distance divided by the width of the thumb metacarpal base described the percentage of radial subluxation of the thumb metacarpal. Radial subluxation was normalized to the width of the thumb metacarpal base and categorized into groups of 0% to 24%, 25% to 49%, 50% to 74%, and 75% to 100% subluxation.

Trapezial space height determination, or the trapezial arthroplasty space, was modified from a previously described technique, which measured the distance between the subchondral surfaces of the thumb metacarpal base and the distal pole of the scaphoid (Fig. A).^30^ Our method is as follows:

1. A line was drawn along the articular surface of the metacarpal base, and another extended perpendicular to the midpoint of this subchondral surface through the axis of the metacarpal, thus defining the distal extent of measurement.
2. An additional line was drawn along the subchondral articular surface of the distal scaphoid, perpendicular to the point of intersection with its long axis, thus defining the proximal extent of measurement.
3. The trapezial space was measured as the perpendicular distance between these two lines along the long axis of the scaphoid and categorized into groups defined by 0–2.4 mm, 2.5–4.9 mm, and 5.0 mm or greater.

Scaphotrapezoid arthritis was evidenced by radiographic signs of OA, including subchondral sclerosis and cyst formation, joint space narrowing, and the presence of marginal osteophytes (Fig. C). Arthritis was recorded as present or absent by location.

Descriptive statistics were used to analyze baseline findings prior to revision surgery, including the patient’s presenting complaints, physical examination, radiographic measurements, and intraoperative revision findings. Radiographic parameters were grouped by evidence of thumb metacarpal instability (radial subluxation, proximal migration, and trapezial space height), scaphotrapezoid arthritis, and MCP joint hyperextension.

## Results

Patients were mostly women (20/23 87.0%) and Caucasian (20/23; 87.0%), with an average age of 57 years (range, 46–70; Table 1). This revision series is derived from 24 index procedures: nine LRTI (one with SwiveLock [Arthrex]), seven suture suspensionplasty (two with SwiveLock), five TightRope (Arthrex), and three partial trapeziectomy. Median time to revision was 14 months (range, 1 month to 10 years; mean, 31 months). Three patients had index partial trapeziectomy. Four patients underwent revision procedures not involving the basal joint; two were second revisions. Only the second revision, which was performed by our surgeons, was counted in this study. One patient underwent lunate and triquetrum excision after inadvertent scaphoid excision during index TightRope procedure, converted 1 year later to LRTI; one had corrective osteotomy of an MCP joint fusion after initial revision of a failed LRTI arthroplasty; and two underwent excision of painful suture material after index suture suspensionplasty. Over the 8 years of this review, 146 primary and four revision arthroplasties originated at our institution for a 2.7% revision arthroplasty prevalence. Inclusive of patients referred specifically for revision surgery, 15% (26/172) of our basal joint procedures were revisions.

**Table 1:** Demographics and Index Revision Operations

Patient Demographics (n = 23)	Values
Female	87.0% (n = 20)
Male	13.0% (n = 3)
Race, White	87.0% (n = 20)
Race, unknown	17.4% (n = 4)
Age, average	57 y
Age, range	46–70 y
Revision Operations (n = 26)	
Time to revision procedure	Median: 14 mo Mean: 31 mo Range: (1 mo–10 y)

Index Basal Joint Procedure (n = 24)	

Index LRTI	37.5% (n = 9)
Full FCR	29.2% (n = 7)
Half FCR	4.2% (n = 1)
Half FCR with swivel-lock device	4.2% (n = 1)
Index suspensionplasty	29.2% (n = 7)
Absorbable suture	8.3% (n = 2)
Nonabsorbable suture	8.3% (n = 2)
Swivel-lock suspensionplasty	8.3% (n = 2)
Weilby tendon sling	4.2% (n = 1)
Index TightRope	20.8% (n = 5)
Index partial trapeziectomy	12.5% (n = 3)
Concurrent carpal tunnel release	12.5% (n = 3)

Nonarthroplasty Reoperation (n = 4)	

Corrective osteotomy MCP joint arthrodesis	1
Proximal row carpectomy	1
Excision of prominent suture	2

FCR, flexor carpi radialis.

### Subjective

In all patients, pain was the principal indication for revision, with nearly half (n = 11; 45.8%) also reporting impaired function. Two thumbs (8.3%) had bothersome dorsal dysesthesias (Table 2), one after index TightRope and another after SwiveLock suspensionplasty.

**Table 2:** Thumb Characteristics at Reoperation

Presenting Complaints Prior to BJA Revision (n = 24)	N (%)		Total, %
Pain	24 (100)		
Impaired function	11 (45.8)		
Dorsal thumb dysesthesias	2 (8.3)		

Physical Examination Findings Prior to BJA Revision (n = 24)			

MCP joint hyperextension	12 (50.0)		
MCP joint hyperextension >30°	5 (20.8)		
Proximal thumb metacarpal subsidence	10 (41.7)		
Other	6 (25.0)		

Radiographic Findings Prior to BJA Revision (n = 23)			

ST arthritis∗	18 (81.8)		
Radial subluxation of thumb metacarpal base∗			
0% to 24% radial subluxation	4 (18.2)		
25% to 49% radial subluxation	8 (36.4)		
50% to 74% radial subluxation	8 (36.4)		82
75% to 100% radial subluxation	2 (9.1)		
Proximal thumb metacarpal migration	17 (73.9)		
Trapezial space height∗			
Trapezial space height 0–2.4 mm	3 (13.6)		73
Trapezial space height 2.5–4.9 mm	13 (59.1)		
Trapezial space height ≥5.0 mm	6 (27.2)		

Intraoperative BJA Revision Findings (n = 24)			

Proximal thumb metacarpal migration	12 (50.0)		
Untreated ST arthritis	9 (37.5)		
Foreign body reaction	6 (25.0)		
MCP joint hyperextension >30 degrees	5 (20.1)		
Impinging osteophytes	4 (16.7)		
Scaphoid-thumb metacarpal impingement	3 (12.5)		
Suture anchor pull-out	2 (8.33)		

Prevalence of presenting complaints, physical examination findings, radiographic disease, and intraoperative revision BJA findings. ST, scaphotrapezoid.

∗ Only 22 thumbs were evaluated for ST arthritis, trapezial space height, and radial subluxation of the thumb metacarpal base (one thumb had inadvertent scaphoid excision and distance could not be measured).

### Physical examination

Twelve thumbs (12/24) exhibited MCP joint hyperextension, which exceeded 30° in five. Ten thumbs (10/24) exhibited painful axial thumb metacarpal instability, in five LRTI and five suspensionplasty procedures. Five patients could not flatten the palm, including three of five index TightRope procedures, one of two index SwiveLock devices (4/7; 57.1%), and one index LRTI with a SwiveLock. Six thumbs exhibited other pathologies, including prior trauma, carpal tunnel syndrome, palpable foreign body (two patients), capitate subluxation, and absent EPL function (Table 2).

### Radiographs

Findings (Table 2) included persistent scaphotrapezoid arthritis (18), greater than 25% radial subluxation of the thumb metacarpal base (18), proximal thumb metacarpal migration (17), and trapezial space less than 5 mm (16). Many patients exhibited multiple radiographic findings; 71% (12/17) of thumbs with proximal subsidence had evidence of untreated scaphotrapezoid arthritis, including all three with index partial trapeziectomy presenting with painful scaphotrapezial arthritis.

### Revision surgery

Intraoperative findings (Table 2) included proximal thumb metacarpal subsidence (n = 12; 50%), untreated scaphotrapezoid arthritis (n = 9; 37.5%), foreign body granuloma (n = 6, 25%), MCP joint hyperextension >30^o^ (n = 5; 20.1%), impinging osteophytes (n = 4; 16.7%), scaphoid-metacarpal impingement (n = 3; 12.5%), and suture anchor pull-out in two thumbs (8.3%). Foreign body granuloma was found in five of eight (62.5%) patients with TightRope or SwiveLock devices. Two-thirds (6/9) of thumbs found to have scaphotrapezoid arthritis intraoperatively exhibited radiographic evidence of the same, whereas only 9 of 18 thumbs suspected of untreated scaphotrapezoid arthritis on prerevision radiographs required partial trapezoid resection at revision surgery.

Thumb metacarpal instability was defined by the presence of either >25% radial subluxation of the metacarpal, proximal thumb metacarpal migration beyond the index metacarpal base, and/or trapezial space height less than 5 mm; 74% (17/23) of thumbs exhibited at least 2, and 26% (6/23) exhibited all three manifestations of thumb metacarpal instability.

## Discussion

The opposable thumb is uniquely human. Perhaps not surprisingly, then, basal joint pain and dysfunction are perceived as being disproportionately impactful on quality of life. Painful thumb dysfunction may lead patients to pursue revision surgery in the relatively uncommon situation of unsuccessful primary BJA. To inform this discussion, we sought to elucidate the common identifiable pathologies encountered during reoperation of primary BJA from a consecutive series of revision procedures at a single institution over a 9-year period.

The prevalence of all-cause revision basal joint procedures originating at our institution was 2.7%, which is consistent with the low published rates of revision following primary BJA.^23^^,^^24^ Among patients seeking revision in this series, painful thumb metacarpal instability and subsidence, untreated scaphotrapezoid arthritis, and MCP joint hyperextension were the most common pathologies evident at reoperation. Additionally, patients receiving TightRope or SwiveLock devices using suture buttons or anchors to stabilize the thumb metacarpal shaft uniquely exhibited painful fixed antepulsion of the thumb and inability to flatten the palm; this iatrogenic dysfunction was likely due to overzealous tightening of the fixed suture device. Painful dysesthesias and foreign body reactions were observed in all but two patients who underwent such procedures. All three patients who underwent hemitrapeziectomy presented with untreated painful radiographic scaphotrapezial arthritis. Four patients required revision after primary BJA that did not involve the basal joint: one corrective osteotomy through a malpositioned hyperflexed MCP joint arthrodesis for rotational instability during pinch, one proximal row carpectomy completion after inadvertent scaphoid excision at primary BJA, and two painful suture knot excisions after suspensionplasty.

Previously reported etiologies of failed BJA include MCP joint hyperextension instability, proximal thumb metacarpal migration, untreated scaphotrapezoid arthritis, subluxation, dislocation, complex regional pain syndrome, neuromas, and infection.^28^^,^^29^ Pain has been identified as an important and prevalent symptom leading to revision BJA.^24^^,^^29^ It was universally present in all patients in our series and accompanied by specific dysfunction in nearly half of patients. Graft extrusion or mechanical failure of the suspension after LRTI as well as scapholunate instability when using the flexor carpi radialis tendon have also been reported.^27^ Our series included nine index LRTI procedures and seven index suspensionplasties; five of each exhibited painful axial metacarpal instability. Likewise, all but two thumbs in this cohort presented with metacarpal instability, manifesting as either radial subluxation, proximal migration, and/or decreased trapezial space height.

A novel finding in this series includes complications related to suture anchors or other synthetic devices. Fixed sutures anchored with TightRope or SwiveLock devices used to secure the thumb metacarpal directly to the index metacarpal were intended to avoid subsidence, but overtightening can result in stiff painful metacarpal convergence, fixed thumb antepulsion, and inability to flatten the palm. Additionally, synthetic devices present their own set of complications, including aseptic loosening, migration or dislodgement, hardware complication, or foreign body reactions, which may yield higher failure rates.^26^^,^^27^ Five of eight “devices,” all of which were synthetic anchors used for direct suture fixation of the thumb to the index metacarpal, were associated with dislodgement at revision surgery and/or foreign body granuloma formation seen on histology. Two patients underwent a revision for painful suture granuloma after suspensionplasty; to avoid this complication, the authors now bury the suture knot beneath the dorsal capsule.

Three-quarters of thumbs were thought to have radiographic evidence of untreated scaphotrapezoid arthritis at presentation, but only half were found to need trapezoid excision at revision surgery. Only two-thirds were found to have scaphotrapezoid arthritis intraoperatively exhibited radiographic evidence of arthritis on prerevision films. All three patients who had index partial trapeziectomy presented with pain and residual arthritis requiring trapezial resection at revision surgery. Intraoperative inspection of retained scaphotrapezoid joint surfaces is essential and facilitated by distraction of the joint, most easily accomplished by spreading the interposed blades of a small scissor. When present, scaphotrapezoid arthritis is addressed by resection of the proximal one-third of the trapezoid with a small osteotome and mallet; tendon interposition seems intuitively desirable, and residual trapezoid instability has not been seen.^31^^,^^32^

Based on the observations from this review, several strategies should guide the revision BJA and may reduce the risk of failed primary procedures. Precise tensioning of the thumb metacarpal suspension is facilitated by temporary K-wire fixation of the thumb metacarpal to the scaphoid in the desired position relative to the index metacarpal. The position of the thumb metacarpal is then adjusted along the axis of the fixation K-wire while holding tension on the suspension material, be it flexor carpi radialis tendon, APL tendon, or synthetic suture, from its index metacarpal insertion. To avoid thumb metacarpal instability, the freshly cut surface of the metacarpal base should rest squarely on the suspension material to ensure the most functional ligament reconstruction.^33^ Metacarpal stabilization can be addressed by a variety of techniques, but both preservation of the trapezial arthroplasty space and mobility of the thumb metacarpal must be provided to achieve optimal function.^34^^,^^35^

Some limitations of this work bear mentioning. The retrospective nature of this review affords identification of associations rather than causes of revision surgery, and the absence of protracted follow-up precludes assessment of outcomes after revision procedures. Although it is unknown if pathologies identified at revision were present at the index procedure, the insidious progression and chronic nature of basal joint arthritis and a median time of only 14 months between index and revision procedures make it highly likely that these pathologies were present at the time of index arthroplasty. Inherent variation in radiographic measurements was mitigated by using a previously reported technique and aggregating three measures of metacarpal instability; internal consistency of these three measures within each thumb supports the findings’ reliability.^30^ Although radiographic assessment might have been optimally performed with other views, the retrospective nature of this analysis limited the availability of specific views; the posteroanterior hand view was used as it was most consistently available across the cohort. Although retrospective studies are subject to selection bias, we captured an unselected consecutive series of patients at a tertiary care center that receives most revision cases in our geographic area with the hope of identifying a representative patient cohort seeking revision surgery. Finally, a small sample size precluded the study of the relationship between specific failure modes and the various primary basal joint surgery techniques.

In summary, awareness of common reasons for reoperation after primary BJA can inform surgical planning for successful revision surgery. Treatment priorities for successful BJA must include stabilization of the thumb metacarpal, recognition of scaphotrapezoid arthritis, and correction of thumb MCP joint instability. Notably, painful inability to flatten the palm following the use of newer fixed suture anchor procedures suggests that metacarpal stabilization must also preserve critical mobility at the base of the thumb, and overtightening of these devices should be avoided to prevent problematic thumb-index metacarpal convergence.

## Conflicts of Interest

No benefits in any form have been received or will be received related directly to this article.
